# Detection of Japanese Encephalitis by Metagenomic Next-Generation Sequencing of Cerebrospinal Fluid: A Case Report and Literature Review

**DOI:** 10.3389/fncel.2022.856512

**Published:** 2022-02-17

**Authors:** Xin Li, Jing Li, Guode Wu, Manxia Wang, Zhang Jing

**Affiliations:** ^1^Department of Neurology, Lanzhou University Second Hospital, Lanzhou, China; ^2^Department of Magnetic Resonance, Lanzhou University Second Hospital, Lanzhou, China

**Keywords:** Japanese encephalitis, central nervous system infection, metagenomic next-generation sequencing, Japanese encephalitis virus, epidemic encephalitis B

## Abstract

Japanese encephalitis (JE) is an acute viral central nervous system disease, although less than 1% of patients infected with Japanese encephalitis virus (JEV) result in JE, which has an extremely poor prognosis. The Routine detection methods for JEV are time-consuming or limited by hospital conditions, therefore, need the quicker and sensitive techniques to detect JEV. Here, we reported a 14-year-old female who was admitted to our hospital with a severe fever, progressively headache and unconsciousness. Based on the clinical presentation, Preliminary diagnosis on admission indicated central nervous system infection of suspected viral meningoencephalitis or autoimmune encephalitis. The patient's symptoms were unrelieved after being treated with empiric antiviral therapy. Magnetic resonance imaging (MRI) showed that the lesions were located in the bilateral thalamus, head of caudate nucleus, and right lenticular nucleus, so we had to consider the possibility of Flaviviruses infection. We sent the cerebrospinal fluid (CSF) for metagenomic next-generation sequencing (mNGS) immediately, subsequent result suggested the infection caused by JEV. Two days later the results of the serum agglutination test confirmed that virus immunoglobulin M antibody positive. After a week treatment with intravenous immunoglobulin (IVIG), meanwhile, the lumbar puncture was used to check the pressure and various indicators of the CSF again to evaluate the treatment effect, An decrease in the number of WBC indicates, protein and unique RNA reads that the previous experimental treatment was effective, accompany by temperature and consciousness of the patient was normalized. Two weeks after admission, the patient was transferred to the rehabilitation hospital, MR showed the lesions had disappeared completely after 2 months of follow-up. We believed that mNGS may be an effective method for rapid identification of JE.

## Introduction

Japanese encephalitis (JE) is the mosquito-borne, an acute viral central nervous system disease in most temperate areas of Asia and Western Pacific, especially in rural or mountain areas where forest and pig farming (Quan et al., 2020). The mortality rate of JE is 20–30% approximately, and 30–50% of survivors have varying degrees of neurological sequelae (Campbell et al., 2011). The prognosis of patients depends on identification early and treatment timely, but the conventional methods such as Enzyme-Linked Immunosorbent Assays (ELISA), Reverse Transcriptase Polymerase Chain Reaction (RT-PCR) were time-consuming or limited by hospital conditions. There is an urgent clinical need for a technology that can quickly and accurately identify pathogens, reduce the risk of death due to delayed treatment. Metagenomics next-generation sequencing (mNGS) is a new detection technology, which has the advantages of fast and precise (Wilson et al., 2019). We report the first case of JE diagnosed by using mNGS of cerebrospinal fluid (CSF).

## Case Report-1

**Figure 1:**
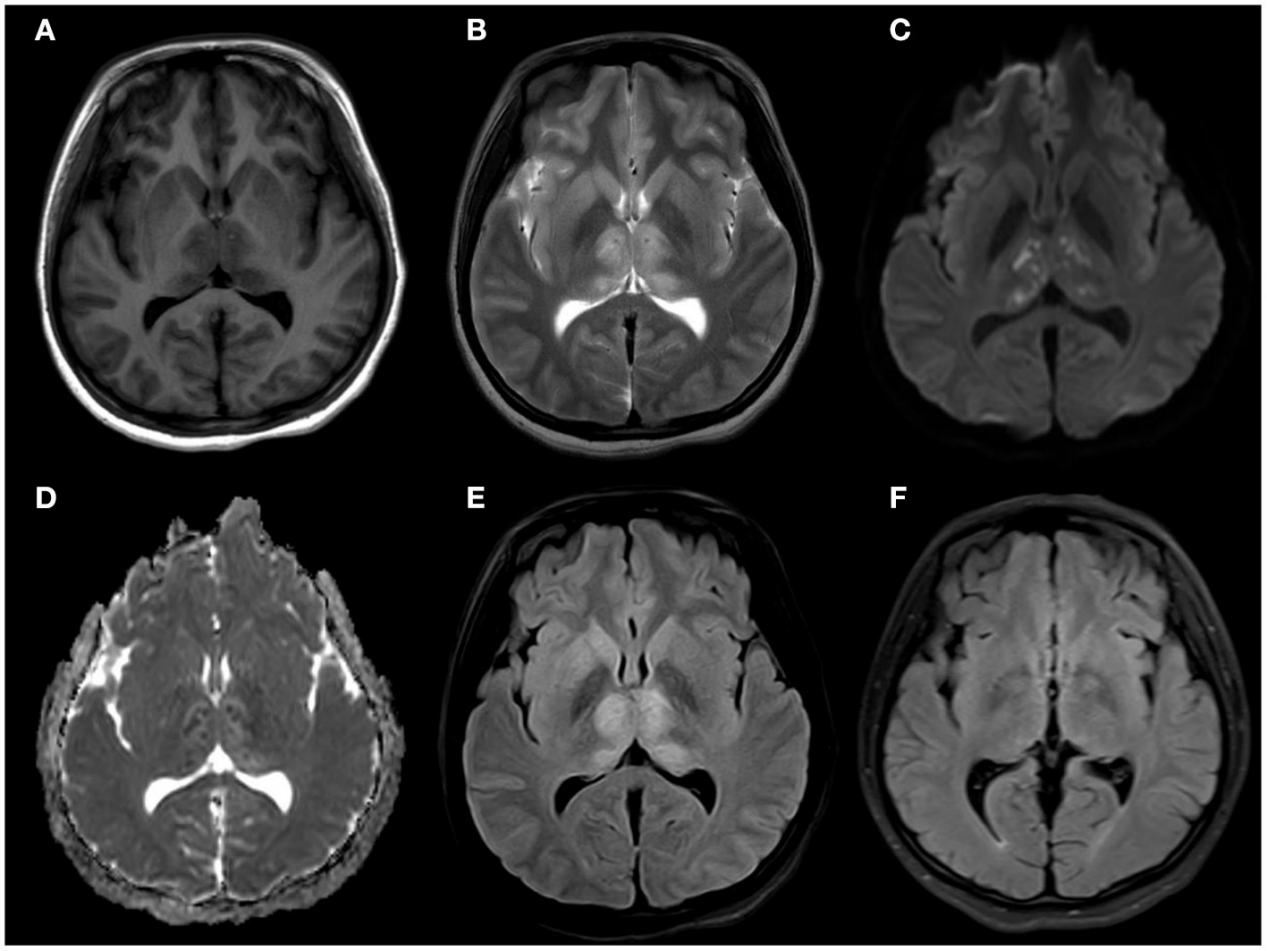
**(A)** MRI brain axial T1: slightly hypointensity in the symmetric thalamus, head of caudate nucleus, and right lenticular nucleus **(B)** MRI brain axial T2: hyperintensity in the symmetric hippocampus, thalamus, head of caudate nucleus, and right lenticular nucleus. **(C,D)** MRI brain axial diffusion-weighted imaging: isointensity bilateral punctate hyperintensity in the symmetric thalamus, and showed hypointensity in ADC. **(E)** MRI brain axial T2 fluid-attenuated inversion recovery: slightly hyperintensity in the symmetric thalamus, head of caudate nucleus, no mass effect. **(F)** MRI brain axial T2 fluid-attenuated inversion recovery: The lesions had disappeared completely after 2 months.

**Figure 2:**
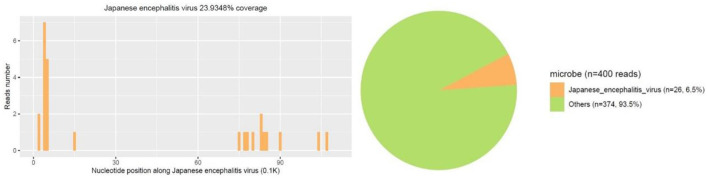
First mNGS result showed a total of 26 unique RNA sequences of Japanese encephalitis virus (JEV), with a coverage of 23.9348% were detected.

**Figure 3:**
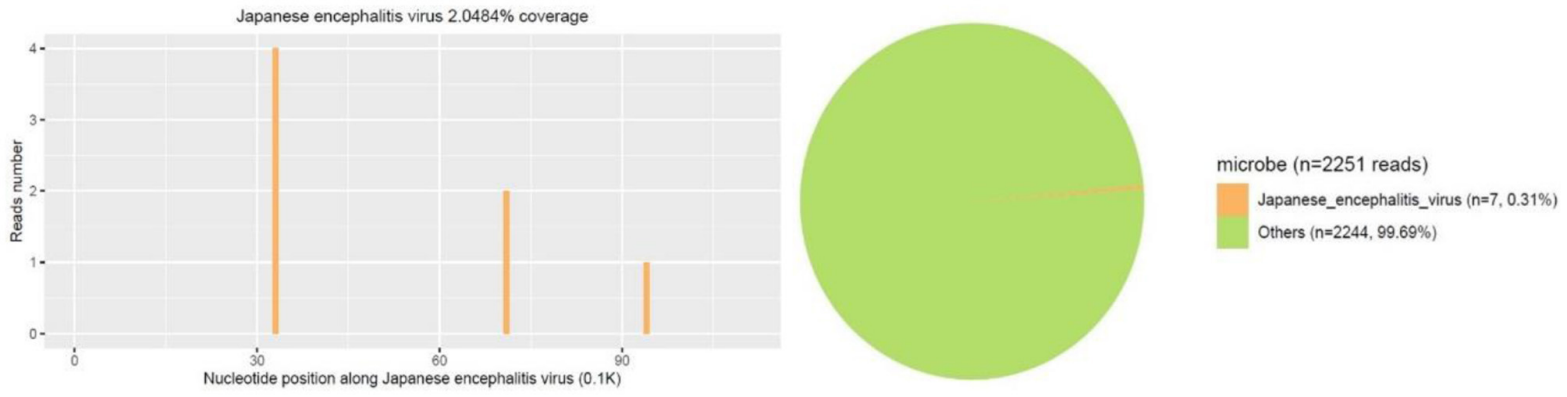
Second mNGS result showed a total of 7 unique RNA sequences of Japanese encephalitis virus (JEV), with a coverage of 2.0484% were detected.

A 14-year-old female patient was admitted to the Second Hospital of Lanzhou University on July 23rd, 2019 due to fever accompanied by progressively deteriorated headache for 3 days, and progressive unconsciousness for 1 day. She developed fever (her body temperature over 40°C) without obvious predisposing factors on July 20th, 2019, accompanied by headache, mainly mild intermittent throbbing pain in the temporal. The symptoms worsened on the next day, and the headache spread to the occipital and back of the neck and lasted longer. Meanwhile, she developed mild fatigue and sore muscles, which were unresponsive to Paracetamol. On the morning of July 22nd, the headache was significantly aggravated obvious, and the duration was prolonged, accompanied by nausea and vomiting. Her sleep increased and she was not easy to wake up that afternoon. When she was taken to our hospital on July 23rd 2019, she was in mild coma. Routine physical examination on admission showed the body temperature of 39.2°C, pulse of 93 beats/min, respiratory rate of 23 times/min, and blood pressure of 124/82 mmHg. Nervous system examination showed consciousness and poor mental state without obvious abnormalities involving high-level cortical functions or cranial nerves. The muscle strength of the limbs was grade 4+. The muscle tension was normal, and tendon reflexes were present without any pathological signs. The neck was rigid, and the mentosternal distance was 4 fingers. Positive Kernig sign and positive Brudzirnki sign were observed. Blood routine on admission plus CRP showed WBC counts at 9.5 × 10^9^/L, neutrophil ratio NE% of 0.43, lymphocyte ratio LY% of 0.57, and CRP of 29 mg/L. The rest laboratory tests showed no abnormalities. Preliminary diagnosis on admission indicated central nervous system infection of suspected viral meningoencephalitis. Acyclovir 0.5 g/8 h, iv, dexamethasone 10 mg, iv. were administered. Brian magnetic resonance imaging (MRI) revealed hyperintensity involving bilateral thalamus, head of caudate nucleus and right lenticular nucleus on July 24th, 2019 (Figures 1A–E). Because of symmetrical thalamic lesions, we have to consider that the patient may suffer from Flaviviruses. Lumbar puncture was subsequently performed with the CSF intracranial pressure of 220 mmH_2_O, WBC counts was 90 × 10^6^/L, of which mononuclear cell accounted for 86.9%, and proteins was 1.07 g/L. In the meantime, syphilis antibodies, autoimmune encephalitis antibodies, rheumatism antibodies, respiratory disease antibodies, and tuberculosis antibodies were all tested for negative. The rest examinations showed no abnormalities. In addition, 8 ml of CSF was collected for mNGS (Accurate Decoding Medical Laboratory, Gansu, China) to determine the pathogen. On July 26, the results of mNGS showed a total of 26 unique RNA sequences of Japanese encephalitis virus (JEV), with a coverage rate of 23.9348% (Figure 2). In order to confirm the JEV infection, the CSF was collected and sent to the Center for Disease Control and Prevention to test the JEV immunoglobulin M antibody. As the patient's condition did not relieve, we used intravenous gamma globulin empirically. On July 28, the patient's JEV immunoglobulin M antibody was positive. Therefore, the diagnosis of JE was finally confirmed. Subsequently, the patient's clinical symptoms improved, her body temperature was basically normal, and her consciousness recovered. A second lumbar puncture was performed to observed cytological and protein changes in CSF and for mNGS detection again on August 1st, 2019. Analysis of CSF showed a decrease in the number of white blood cells and protein levels (WBC count was 42 × 10^6^/L, of which monocyte percentage for 56.9%, and protein was 0.57 g/L). The result of mNGS detected seven the unique RNA reads of JEV and genome coverage 2.0484% on August 3rd, 2019 (Figure 3), which was significantly better than the previous results on July 26th, 2019. After pure antiviral treatment for 5 days, the patient was transferred to the rehabilitation hospital on August 5th, 2019. Follow-up MRI showed that the lesions disappeared completely after2 months, on October 2nd, 2019 (Figure 1F).

**Figure 4:**
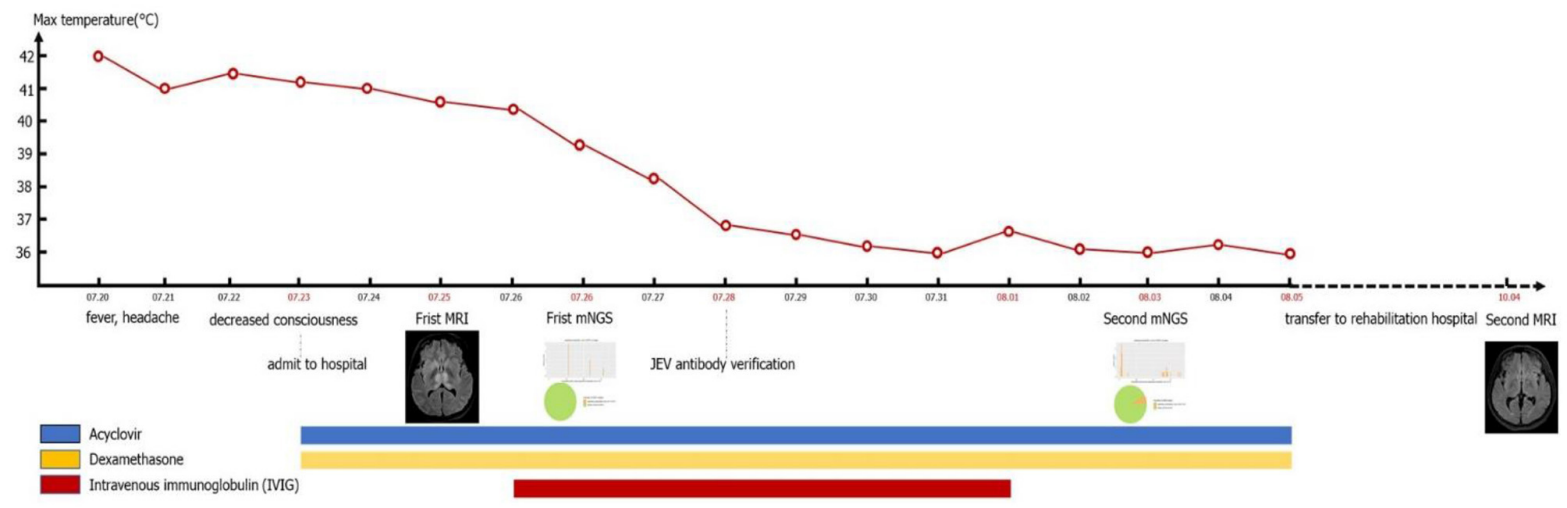
Timeline of the patient's clinical symptoms, temperature change process, drug use, mNGS, and MRI.

Through timeline of the patient's clinical symptoms, temperature change process, drug use, mNGS and MRI (Figure 4), we can see the patient's treatment process in detail.

## Discussion

Japanese encephalitis is also known as epidemic encephalitis B, an acute mosquito-borne viral central nervous system infection caused by JEV, which is mainly prevalent in Asia and the Pacific. The WHO estimates that approximately 67,900 cases of JE are distributed in these epidemic areas, and the incidence rate is about 1.8/10,000 (Solomon, 2006; Wang and Liang, 2015). However, there is a trend that epidemic areas expand (Connor and Bunn, 2017). In 2010, the nucleic acid sequence of the JEV-NS5 segment was detected in the specimens of *Culex pipiens* pollens and birds in northeastern Italy (Platonov et al., 2012; Zeller, 2012). In Africa, JEV nucleic acid was detected from Fiebre amarilla patients through deep sequencing (Simon-Loriere et al., 2017).

A variety of animals can be infected with JEV, including pigs, horses, cattle, sheep, and birds. Among them, pigs are very susceptible to JEV (Le Flohic et al., 2013; Mansfield et al., 2017). Pigs can maintain a state of high-level viremia for a long time after infection. They are the main host and important source of infection for JEV (van den Hurk et al., 2009). Mosquito bite is the main mode of transmission of JE. At present, more than 30 species of mosquitoes are known to be vectors of JE, and the type and number of mosquitoes can affect the incidence and prevalence of JE (Le Flohic et al., 2013). *Culex tritaeniorhynchus* is widely distributed among mosquito vectors, and is highly susceptible to JE. It is the most important mosquito species in the transmission of JE (Ghosh and Basu, 2009; Hsieh and St John, 2020). Japanese encephalitis virus (genus Flavivirus; family: Flaviviridae) is a forward single-stranded RNA virus containing an envelope, which is neuroinvasive and neurotoxic in humans (Impoinvil et al., 2011). After humans are bitten by mosquitoes carrying JEV, the virus enters the local skin and lymph nodes through receptor-mediated endocytosis and is replicated in monocyte-macrophages and dendritic cells, causing transient low-level viremia (Banerjee and Tripathi, 2019; Filgueira and Lannes, 2019). Infected cells cross the blood-brain barrier (BBB) through endothelial cell phagocytosis, and induce the production of inflammatory cytokines and chemokines, leading to the destruction of the BBB (Miner and Diamond, 2016; Patabendige et al., 2018; Hsieh and St John, 2020). It is worth noting that the destruction of the BBB is not caused by the virus itself. Inflammatory cytokines or chemokines can reduce the permeability of the BBB by down-regulating tight junction proteins. At the same time, inflammatory cytokines can further impair the BBB by inducing the expression of adhesion molecules on BBB endothelial cells (Chen et al., 2014; Chang et al., 2015). Then the infected cells migrate from the periphery to the central nervous system, trigger humor and cell-mediated immune responses and induce the expression of pro-inflammatory cytokines. The activation of microglia will lead to the overproduction of pro-inflammatory mediators and cause extensive destruction of neurons and symptoms of acute encephalitis (Lannes et al., 2017; Ashraf et al., 2021). The clinical manifestations and prognosis of patients depend on the virulence of the virus and the host immune response. The main clinical manifestations of patients include headache, high fever, disturbance of consciousness, seizures, and even respiratory failure (Basumatary et al., 2013). Rare manifestations include mandibular-facial nerve twisting tremor (Takeuchi et al., 2014), cervical dystonic tremor (Spagnolo et al., 2014), phrenic nerve palsy (Chaudhuri et al., 2021), and upper and mixed upper and lower motor neuron damage (Ghosh et al., 2020). Japanese encephalitis is often secondary to a variety of diseases including Guillain-Barré syndrome (Xiang et al., 2014; Wang et al., 2020), Secondary Parkinson's disease (Tadokoro et al., 2018), anti-N-methyl-D-aspartate receptor encephalitis (Tian et al., 2019; Wang et al., 2020), acute flaccid myelitis (Dev et al., 2020; Shen et al., 2020), cerebral venous sinus thrombosis (Jia et al., 2012) and so on. The patient in this case had typical high fever, headache, and hypoconsciousness, and the clinical symptoms are consistent with the typical clinical manifestations of JE. The imaging manifestations of JE are extensive involvement of the brain and spinal cord. Among which the thalamus and midbrain are the most severely affected, the parietal lobe, frontal lobe, and hippocampus of the brain are significantly affected, and the spinal cord is the least affected. Symmetry of thalamic lesions is the characteristic of JE (Prakash et al., 2004; Handique and Barkataky, 2008; Lai et al., 2008). Magnetic resonance imaging of this case showed lesions in the bilateral thalamus, the head of the caudate nucleus, and the right lenticular nucleus. The lesions showed slightly low signal intensity on T1WI, slightly higher signal intensity on T2WI, and high signal intensity on FLAIR sequence, which is consistent with changes in acute encephalitis. The DWI sequence showed mainly iso-signal intensity mixed with patchy hyperintensity, suggesting that the lesions were mainly vasogenic edema, and a few showed cytotoxic edema. The imaging changes are related to the pathological mechanism of the patient's disease process, and the spot-to-uniform pattern of cytotoxic edema may reflect the pattern of virus replication in the thalamus (Arahata et al., 2019). It was confirmed in animal experiments that the RNA of JEV mainly accumulates in the thalamus and basal ganglia, indicating that the virus replicates specifically in these areas (Ricklin et al., 2016). Although Neuroimaging features of JE have showed that involvement of temporal lobe and hippocampus is also one of the characteristics in previous study (Handique et al., 2006), the patient in this case did not have hippocampal involvement, and there were no clinically related mental abnormalities and memory impairment, which may be related to her mild condition. Through the characteristics of imaging, we suspected the possibility of flavivirus infection, but due to technical restriction, we can't detect the specific virus type by PCR. After weighing and analyzing the benefits of treatment and the risk of serious consequences of JEV, before completing the confirmatory test of JEV antibodies, we conducted experimental treatment immediately based on the results of mNGS. Although there is no special treatment for JE, early supportive treatment can limit the development of neurological sequelae. Based on previous clinical experience, the patient used antiviral drugs (aciclovir), dexamethasone, and experimentally used intravenous gamma globulin for 5 days. The treatment effect was evaluated based on the unique RNA readings in the CSF samples on July 26, 2019 and August 7, 2019. After 10 days of treatment, the number of JEV readings was reduced from 24 to 7. Fortunately, after 20 days of hospitalization, the patient's condition gradually stabilized and she was transferred to the rehabilitation hospital. A follow-up MRI showed that the lesions disappeared completely after 2 months. As a new detection method, mNGS does not rely on traditional microbial culture and directly performs high-throughput sequencing of nucleic acids in samples. It can quickly and indiscriminately detect pathogenic microorganisms in samples (Salzberg et al., 2016; Gu et al., 2019; Wilson et al., 2019), such as bacteria, viruses, fungi, and parasites, to the difficult infections or rare pathogenic microorganisms especially (Fan et al., 2020; Cao et al., 2021; Liu et al., 2021).

Current next-generation sequencing (NGS) are widely utilized for pathogen identification, but they can only sequence DNA molecules in most cases. While DNA composes the genetic material for pathogenic bacteria and fungi, RNA viruses compose a large fraction of infectious pathogens in central nervous system infectionas well, Flavivirus (including West Nile virus, JE virus and so on), severe fever with thrombocytopenia syndrome virus (SFTSV), for example. It is noteworthy that the severe acute respiratory syndrome coronavirus 2 (SARS-CoV-2) responsible for the coronavirus disease 2019 (COVID-19) which belongs to RNA virus (Manso et al., 2017; Gao, 2018; Wang et al., 2020a,b). Thus, RNA viruses will be neglected from solely DNA-based mNGS, sometimes hindering important diagnoses. Due to the cost of medical economics, mNGS is rarely used for simultaneous detection of RNA and DNA of pathogenic microorganisms in clinical diagnosis, our experience is that when the DNA sequence of pathogenic microorganism is not detected and the treatment effect of the patient is poor, or the patient lives in the epidemic area, or the encephalitis patients with typical symmetrical basal ganglia lesions can directly choose mNGS for RNA detection, or DNA and RNA can be detected together. Although mNGS still has some problems to be solved, such as distinguishing infection from colonization, identifying exogenous nucleic acid pollution, the targeted sequencing are overcoming these defects (Simner et al., 2018).

As far as we know, this is the first clinical case of JE that has been diagnosed using mNGS. We must emphasize the potential value of mNGS in the identification of pathogens of central nervous system infection, because it has the advantages of sensitivity, speediness and accuracy.

## Data Availability Statement

The raw data supporting the conclusions of this article will be made available by the authors, without undue reservation.

## Ethics Statement

The studies involving human participants were reviewed and approved by Lanzhou University Second Hospital Ethics Committee. The patients/participants provided their written informed consent to participate in this study. Written informed consent was obtained from the individual(s) for the publication of any potentially identifiable images or data included in this article.

## Author Contributions

XL and JL contributed to the study design and drafted the manuscript. MW contributed to PCR validation of the data in the literature and edited the manuscript. ZJ contributed to MRI of the data in the literature. GW contributed to supervised writing of the manuscript and critically reviewed the paper. All authors contributed to the article and approved the submitted version.

## Funding

This work was supported by grants from the Natural Science Foundation of Gansu Province, China (Grant No. 21JR1RA136).

## Conflict of Interest

The authors declare that the research was conducted in the absence of any commercial or financial relationships that could be construed as a potential conflict of interest.

## Publisher's Note

All claims expressed in this article are solely those of the authors and do not necessarily represent those of their affiliated organizations, or those of the publisher, the editors and the reviewers. Any product that may be evaluated in this article, or claim that may be made by its manufacturer, is not guaranteed or endorsed by the publisher.
